# Treatment of secondary central nervous system involvement in systemic aggressive B cell lymphoma using R-MIADD chemotherapy: a single-center study

**DOI:** 10.1186/s41016-021-00238-0

**Published:** 2021-04-02

**Authors:** Yuchen Wu, Xuefei Sun, Xueyan Bai, Jun Qian, Hong Zhu, Qu Cui, Ruixian Xing, Yuedan Chen, Qing Liu, Wenyuan Lai, Junhong Li, Yaming Wang, Shengjun Sun, Nan Ji, Yuanbo Liu

**Affiliations:** 1 Department of Hematology, Beijing Tiantan Hospital, Capital Medical University, Beijing, China; 2 Department of Neurosurgery, Beijing Tiantan Hospital, Capital Medical University, Beijing, China; 3 Department of Neurosurgery, Beijing Xuanwu Hospital, Capital Medical University, Beijing, China; 4 Neuroimaging Center, Beijing Tiantan Hospital, Capital Medical University, Beijing, China

**Keywords:** Central nervous system, B cell lymphoma, SCNSL, R-MIADD, Chemotherapy

## Abstract

**Background:**

Secondary central nervous system lymphoma (SCNSL) is defined as lymphoma involvement within the central nervous system (CNS) that originated elsewhere, or a CNS relapse of systemic lymphoma. Prognosis of SCNSL is poor and the most appropriate treatment is still undetermined.

**Methods:**

We conducted a retrospective study to assess the feasibility of an R-MIADD (rituximab, high-dose methotrexate, ifosfamide, cytarabine, liposomal formulation of doxorubicin, and dexamethasone) regimen for SCNSL patients.

**Results:**

Nineteen patients with newly diagnosed CNS lesions were selected, with a median age of 58 (range 20 to 72) years. Out of 19 patients, 11 (57.9%) achieved complete remission (CR) and 2 (10.5%) achieved partial remission (PR); the overall response rate was 68.4%. The median progression-free survival after CNS involvement was 28.0 months (95% confidence interval 11.0–44.9), and the median overall survival after CNS involvement was 34.5 months. Treatment-related death occurred in one patient (5.3%).

**Conclusions:**

These single-centered data underscore the feasibility of an R-MIADD regimen as the induction therapy of SCNSL, further investigation is warranted.

## Background

Secondary central nervous system lymphoma (SCNSL) refers to secondary involvement of the brain, eye, spine, meningeal by systemic lymphoma [1, 2]. It is a devastating complication of systemic lymphoma, occurring in 5–10% of diffuse large B cell lymphoma (DLBCL) patients with very few long-term survivors under conventional treatment [3]. SCNSL can occur in combination with systemic disease or present as an isolated relapse. The prognosis of SCNSL is poorer than primary central nervous system lymphoma (PCNSL); in the largest cohort of SCNSL to date, less than half of the patients reached CR by the end of the induction treatment, and the median overall survival (OS) post-CNS involvement was only 3.9 months [4]. Therefore, further explorations on the treatment of SCNSL, especially initial induction, are needed to improve CR rate and outcomes.

Treatment of SNCSL requires eradication of both the systemic and CNS disease. Standard first-line chemotherapy for systemic disease has limited efficacy in treating patients with CNSL [5]. High-dose methotrexate (HD-MTX)-based regimens are the most commonly used therapy in CNSL for their CNS penetration ability. HD-MTX in combination with high-dose cytarabine can improve survival significantly in patients with PCNSL [6]. Procarbazine, etoposide, ifosfamide, thiotepa, carmustine, and other drugs which can cross the blood-brain barrier have also been included in combination with HD-MTX and/or cytarabine to further improve outcomes [7]. Doxorubicin is not included in regimens for treating PCNSL because of its inability to penetrate the blood-brain barrier [8]. However, with an alternative liposomal formulation, doxorubicin has proved to be a promising drug for CNSL in several studies [9]. In addition, high-dose chemotherapy followed by autologous stem cell transplantation (ASCT) was introduced and showed a beneficial effect. However, ASCT has been offered to only a small number of younger patients but elicited a favorable response .Therefore, several studies have explored the effectiveness of non-transplant regimens for SCNSL and indicate that this could be an effective treatment for patients with SCNSL [10–12]. With this information in mind, we proceeded to treat SCNSL patients with the combination of rituximab, HD-MTX, ifosfamide, cytarabine, liposomal formulation of doxorubicin, or an R-MIADD regimen and carried out a retrospective study to explore the effectiveness and tolerance of this novel chemotherapeutic combination.

## Methods

Clinical data of a total of 19 SCNSL patients were retrospectively reviewed using electronical medical records at the Department of Hematology at Beijing Tiantan Hospital, Capital Medical University, from January 2015 to August 2019. Database was approved by Beijing Tiantan Hospital Ethics Committee; all patients gave written informed consent. Inclusion criteria were as follows: histologic diagnosis of DLBCL or mantle cell lymphoma with blast variant; CNS involvement (including brain and/or meninges and/or cranial nerves and/or eyes and/or spinal cord) determined using stereotactic biopsy, cerebrospinal fluid, cytology/flow cytometry, or brain magnetic resonance imaging (MRI) at diagnosis, or relapse after conventional chemoimmunotherapy; aged 18–70; and an Eastern Cooperative Oncology Group performance status ≤ 3. Patients with primary CNS lymphoma, hepatitis B surface antigen positivity, anti-hepatitis C virus serologic positivity, human immunodeficiency virus, or other immunodeficiency diseases were excluded. Patients were treated with an R-MAIDD regimen in 21-day cycles, specifically the following: rituximab 375 mg/m^2^ infusion on day 0, HD-MTX 3.5 g/m^2^ infusion within 3.5 h on day 1 (with folinic acid rescue), ifosfamide 1.2 g/m^2^ infusion on day 2, cytarabine 1 g/m^2^ infusion on day 3, dexamethasone 10 mg intravenously on days 1–3, and liposomal doxorubicin infusion 20–25 mg/m^2^ on day 4. Patients who did not achieve CR proceeded to have salvage treatment of whole brain radiotherapy with 36 Gy and a boost to tumor bed for a total of 40–50 Gy. All patients received either post-R-MIADD regimen CNS radiotherapy or consolidation chemotherapy according to clinical response.

Response assessment was performed within 4 weeks of the final R-MIADD chemotherapy. Enhanced MRI and whole-body CT with positron emission tomography scan were used for assessment. Treatment response of systemic lymphoma was graded according to the 2014 Lugano criteria. Clinical response in CNS disease was assessed using the International Workshop to Standardize Baseline Evaluation and Response Criteria for Primary CNS Lymphoma [13], and MRIs of all patients were assessed by two experienced neuroradiologists. Toxicities were assessed and recorded using the Common Terminology Criteria for Adverse Events Version 3.0 [14]. Post-CNS involvement OS was defined as the time from initiation of the CNS disease to the last follow-up or death from any cause. Post-CNS involvement progression-free survival (PFS) was calculated from initiation of the CNS disease to disease progression or last follow-up. Survival analyses were performed using the Kaplan-Meier method with SPSS Statistics, Version 24.0. There were not enough subjects to perform reliable multivariate analysis. All tests were two-sided, and a *p* value < 0.05 was considered statistically significant.

## Results

### Clinical characteristics of systemic disease in SCNSL patients

The median age at onset of the systemic disease was 58 (20–72) years (Table 1). Among the 19 SCNSL patients, 10 (52.6%) had simultaneous involvement both inside and outside of the CNS when initially diagnosed, defined as “new disease;” five (26.3%) had CNS disease in the latter part or within 3 months of completing primary therapy, defined as “refractory disease;” and four (21.1%) had a CNS relapse that may have been combined with systemic disease, defined as “relapse disease.” Extranodal involvement as the initial systemic disease was observed in eight patients (breasts, *n* = 3; testis, *n* = 2; bone marrow, *n* = 1; prostate, *n* = 1; and stomach, *n* = 1). The histological findings were DLBCL in 18 (94.7%) patients, and mantle cell lymphoma was diagnosed in one (15.3%) patient. Fourteen (73.7%) patients were graded as Ann Arbor staging III–IV at initial diagnosis.

**Table 1:** Clinical characteristics of systemic disease in SCNSL patients

Characteristics	*N*	%
Age		
Median (range)	58 (20–72)	
≤ 60	10	52.6
> 60	9	47.4
Gender		
Male	9	47.4
Female	10	52.6
Initial disease location		
Breasts	3	15.8
Testis	2	10.5
Lymph node	11	57.9
Others	3	15.9
Ann Arbor stage		
I–II	5	26.3
III–IV	14	73.7
Treatment of initial disease		
R-CHOP like regimen	9	47.4
Other regimen	10	52.6
CNS prophylaxis		
Yes	2	10.5
No	17	89.5

For treatment after onset of systemic disease, nine (47.7%) patients received R-CHOP or similar regimens and two of these patients received intrathecal injection of methotrexate as CNS involvement prophylaxis. One patient received ASCT after achieving CR of systemic disease and developed CNS disease afterward. Ten patients with new SCNSL received CNS-targeting treatment, which is described later in this section.

### Clinical characteristics of CNS disease in SCNSL patients

The median age at onset of CNS disease was 59 (20–76) years (Table 2). The most common symptoms at the initial stage of CNS disease were increased intracranial pressure symptoms and dizziness, seen in five (26.3%) of the SCNSL patients. Other symptoms included blurred vision (*n* = 2), limb weakness (*n* = 2), seizure (*n* = 1), somnolence (*n* = 1), and focal neurological deficits (*n* = 3). Regarding radiological features, brain parenchymal lesions were found in all 19 patients. Single lesions were noted in six (31.6%) patients, and 13 (68.4%) patients had multiple lesions; 10 (47.4%) patients had tumors involving the deep part of the brain, including the cerebellum, basal ganglia, corpus callosum, and brain stem.

**Table 2:** Clinical characteristics of CNS disease in SCNSL patients

CNS characteristics	*N*	%
CNS age		
Median, range	59, 20–76	
≤ 60	10	52.6
> 60	9	47.4
Initial symptoms		
Headache	5	26.3
Dizziness	5	26.3
Blurred version	2	10.5
Limb weakness	2	10.5
Seizure	1	5.3
Somnolence	1	5.3
Focal neurological deficits	3	15.8
ECOG-PS		
≤ 1	6	31.6
> 1	13	68.4
Type of CNS relapse		
New disease	10	52.6
Relapse	4	21.1
CNS only	3	
Combined CNS and systemic disease	1	
Refractory	5	26.3
Multiplicity		
Single	6	31.6
Multiple	13	68.4
Enhancement		
Homogenous	11	57.9
Patchy	7	36.8
None	1	5.3
Location of disease		
White matter	16	84.2
Cerebellum	3	74.2
Brain stem	3	15.8
Deep gray matter	10	47.4
Diagnosis approaches		
Biopsy	10	52.6
Surgery section	4	21.1
Enhanced MRI	4	21.1
CSF cytology	1	5.3
Histology at relapse		
ABC	10	52.6
GCB	4	21.1
NA	5	26.3

*ABC* activated B cell, *GCB* germinal center B cell, *NA* not available

Four (21.1%) patients were diagnosed with typical radiological manifestations on enhanced MRI, 10 (52.6%) patients were diagnosed through stereotactic biopsy, four (21.1%) patients underwent tumor resection, and one (5.3%) patient was diagnosed using cerebrospinal fluid cytology. Histologically, four (4/10) cases showed germinal center B cell phenotypes (CD10 + BCL-6 +/− MUM-1+/−), and 10 (10/14) cases showed peripherally activated B cell phenotypes (CD10-BCL- 6 +/− MUM-1+ or CD10-BCL-6-MUM-l).

### Treatment and responses

**Fig. 1:**
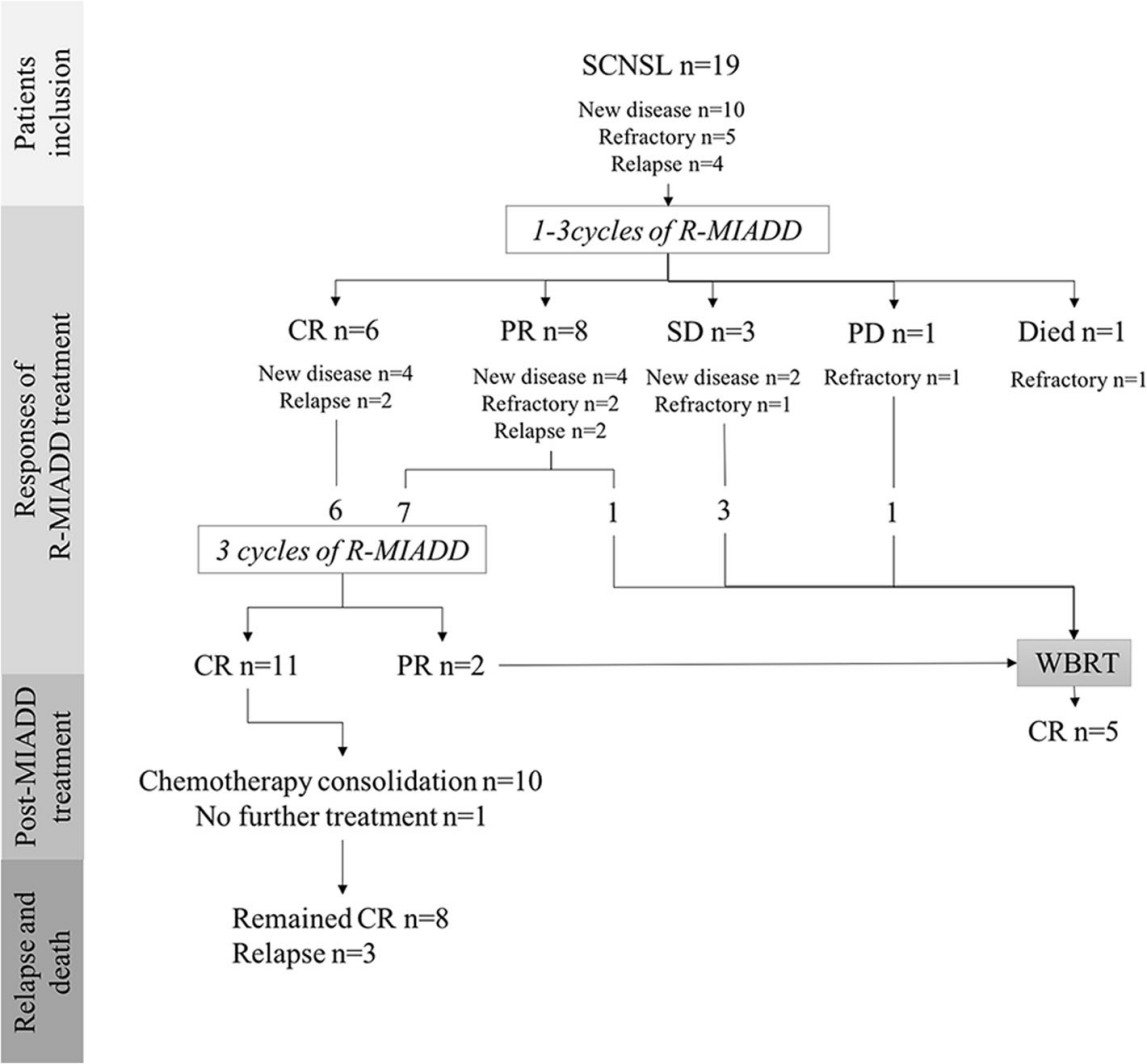
Course of therapy, responses, and clinical outcomes. CR, complete response; PR, partial response; PD, progressive disease; SD, stable disease; WBRT, whole brain radiotherapy

All patients were treated with R-MIADD regimen, one with cerebrospinal fluid dissemination received intrathecal MTX (MTX 10 mg, dexamethasone 5 mg) simultaneously. After 1–3 cycles of induction, six patients reached CR, eight patients were assessed as PR, three had stable disease, and one patient had disease progression (DP) (Fig. 1). One patient died of neutropenic sepsis after the second cycle of chemotherapy.

All six patients with CR and seven patients with PR continued R-MIADD treatment. By the end of the induction treatment, 11 patients attained CR and 2 attained PR (Table 3). Patients with stable disease and DP proceeded with whole brain radiotherapy (WBRT), and one patient with PR also turned to WBRT because of financial difficulties. All five patients achieved CR after WBRT.

**Table 3:** Response of SCNSL patients after induction therapy

Response	Total		New disease		Refractory disease		Relapse disease	
	*N* = 19	%	*N* = 10	%	*N* = 5	%	*N* = 4	%
CR	11	57.9	7	70	2	40	2	50
PR	2	10.5	1	10	0	0	1	25
WBRT*	5	26.3	2	20	2	40	1	25
Death	1	5.3	0	0	1	20	0	0

*CR* complete remission, *PR* partial remission, *SD* stable disease, *PD* progression disease, *WBRT* whole brain radiotherapy

*WBRT indicated SCNSL who turned to WBRT during the induction treatment

### Progression-free and overall survival

By the end of the induction treatment, 11 (57.9%) patients achieved CR and 2 (10.5%) patients achieved PR for an overall response rate (ORR) of 68.4%. Of the 10 “new disease” patients, 7 attained CR and 1 attained PR. For “refractory” and “relapse” patients, ORR was 40% (CR 40%) and 75% (CR 50%), respectively.

**Fig. 2:**
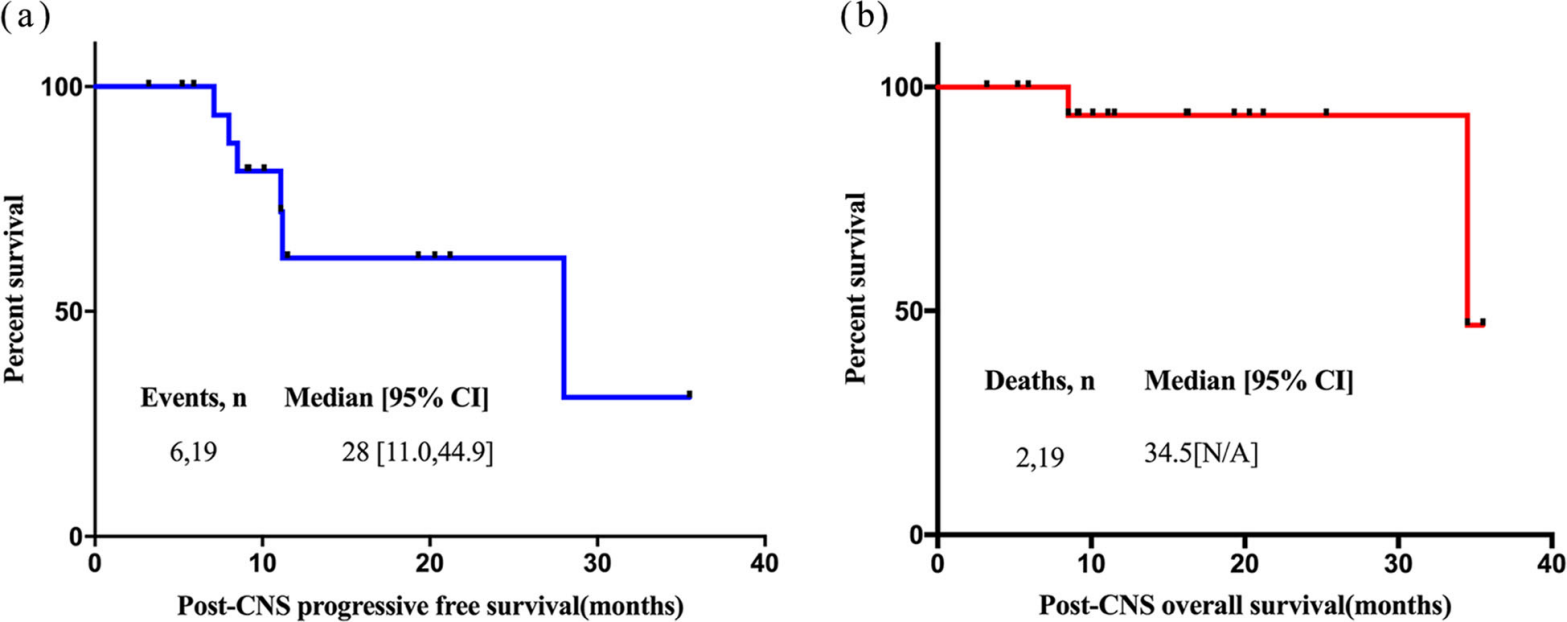
**a** Post-CNS progression-free survival of 19 SCNSL patients. **b** Post-CNS Overall survival of 19 SCNSL patients

The median follow-up time after the onset of CNS disease was 11.1 (3.2–35.5) months, the median post-CNS involvement PFS was 28.0 months (95% CI 11.0–44.9) (Fig. 2a), and the post-CNS OS was 34.5 months (95% CI N/A) (Fig. 2b).

### Post-R-MIADD regimen treatment

After achieving CR, 10 patients continued with chemotherapy for consolidation treatment with a combination of ifosfamide, etoposide, and cytarabine every 3 months. One patient did not receive further treatment. The five patients who did not respond to the R-MIADD regimen proceeded with WBRT and all of them attained CR. After CR, these patients proceeded with ifosfamide, etoposide and cytarabine consolidation every 3 months.

### Toxicity, relapses, and cause of death

Hematologic toxicity (16 [84.2%] patients) was most the common adverse effect of the treatment, with 3 (15.8%) patients experiencing grade 4 myelosuppression. The number of patients with grade 3–4 myelosuppression with either isolated relapse or concurrent disease was 2 (20%) and 6 (66.7%), respectively, although without statistical difference (*p* = 0.07). Elevated aminotransferase levels were observed in 8 (42.1%) patients, and elevated bilirubin levels were found in 2 (10.5%) patients. No severe mucosa damage was observed during the treatment. Two (10.5%) patients experienced temporarily elevated creatinine after MTX administration, but their renal function returned to normal after urine alkalinization and hydration accelerated the excretion of MTX.

**Fig. 3:**
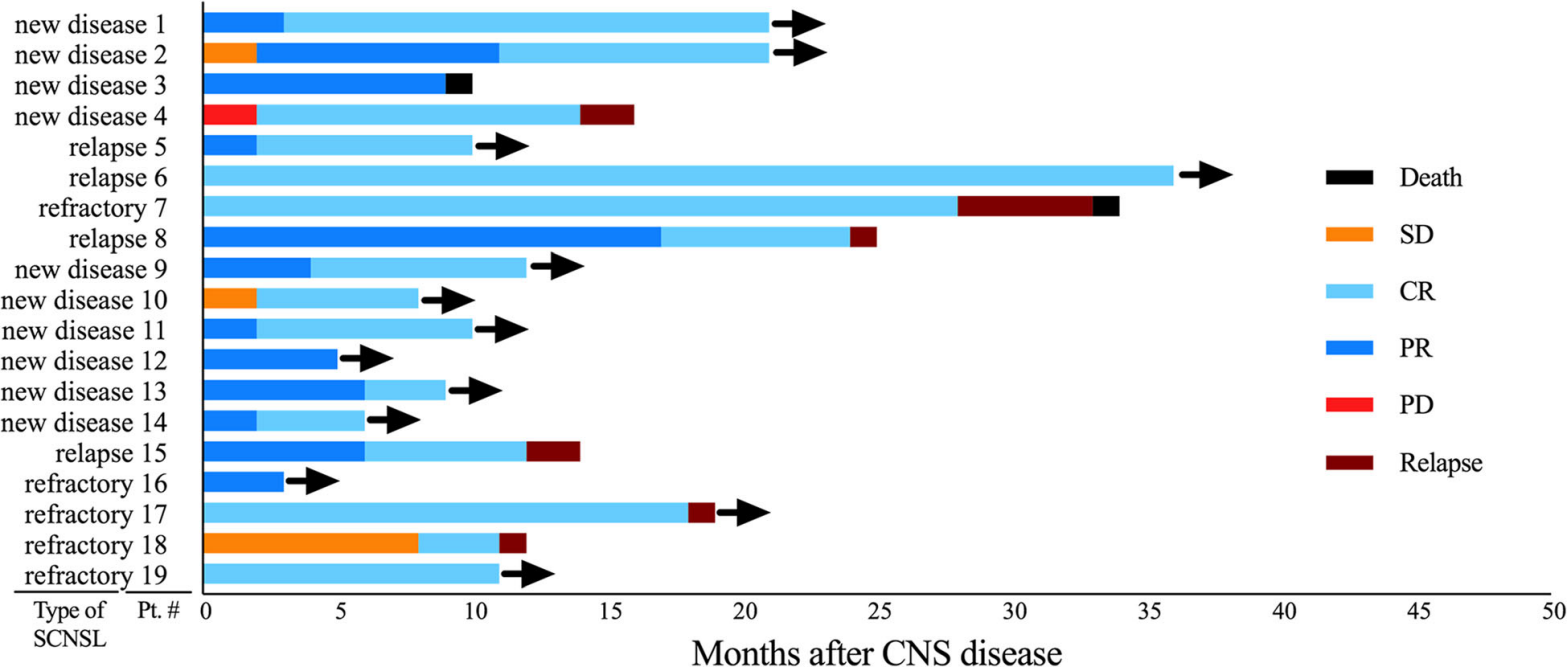
Swim lane plot of treatment duration and response for all patients. CR, complete response; PR, partial response; PD, progressive disease; SD, stable disease

One patient with refractory disease died during the induction treatment because of myelosuppression and severe pneumonia after chemotherapy, which resulted in toxic shock syndrome. Of the 11 patients who obtained CR with the R-MIADD regimen alone, three had isolated relapses in the CNS (two “new disease” and one “relapse disease” patients); one relapse occurred in the spine and two relapses occurred in brain parenchyma; and one patient died of CNS DP (Fig. 3).

## Discussion

We reported the combination of an R-MAIDD regimen to treat SCNSL patients resulting in an ORR of 68.4% and a post-CNS involvement PFS of 28.0 months (95% CI 11.0–44.9). These results indicate that a non-transplant regimen of R-MIADD could potentially be an effective treatment for SCNSL.

There is currently no randomized study that defines the optimal regimen to treat SCNSL because of the rarity of this disease. Few prospective studies favoring ASCT have demonstrated improved outcome in young and fit SCNSL patients [15–19]. In a prospective study undertaken by Korfel et al., 30 SCNSL patients were treated with intravenous HD-MTX, ifosfamide, and dexamethasone with intrathecal liposomal cytarabine; patients that did not respond to this combination were then given thiotepa and cytarabine for the second cycle, while patients that did respond to it received thiotepa, carmustine, and an etoposide regimen-conditioned ASCT with a CR response of 50% and PR 7% [16]. Ferreri et al. implemented a regimen that included high doses of MTX and cytarabine, followed by rituximab, cyclophosphamide, cytarabine +/− etoposide targeting residual systemic disease prior to ASCT, and carmustine, etoposide, cytarabine, and melphalan conditioned ASCT. In patients that responded to this treatment, the CR rate was 63% with a 2-year event-free survival rate of 50% [15]. Despite the encouraging results of the chemoresponsive patients, the majority of patients with SCNSL could not proceeded to ASCT because of poor response to the initial treatment. In a large international cohort study that included 291 cases of secondary CNS involvement in DLBCL, 173 patients received systemic chemotherapy and, of these, only 25 patients received high-dose chemotherapy followed by ASCT. Moreover, the study results indicated that for patients in CR after initial therapy, ASCT consolidation did not prolong OS in cases of isolated SCNSL [4].

Therefore, several studies turned to non-transplant regimens for SCNSL and focused on improving the response rate to initial treatment. Nijland et al. used methotrexate, etoposide, carmustine, and methylprednisolone combined with R-CHOP as induction therapy. After achieving remission, patients underwent whole brain radiotherapy for consolidation. The complete response rate was 57%, with 3 years PFS and an OS rate of 45% (95% CI 34–56%) and 49% (95% CI 38–60%), respectively [10]. Those findings are comparable to the figures reported herein. In another pilot study, Chihara et al. treated eight patients with DA-EPOCH-R combined with HD-MTX, and all eight patients achieved CR [11]. Nagle et al. reported HD-MTX combined with temozolomide for the treatment of SCNSL resulted in the median OS of 4.8 months and 4-year OS of 25% [12].

The regimen used in our study contained HD-MTX, cytarabine, and ifosfamide, in addition to liposomal doxorubicin, but the doses administered in our regimen were lower than those administered the study mentioned previously. Our approach led to better treatment response without additional treatment toxicity. By the end of the induction therapy, 57.9% of patients achieved CR and post-CNS PFS was 28.0 months (95% CI 11.0–44.9), which is comparable to an ASCT-containing regimen. Substantially longer survival than the median PFS was seen in two patients (Patient 6 and 7) at the ages of 39 and 20 years. Previous reports indicate that long-term survival was seen in a small, albeit clinically relevant, proportion of younger SCNSL patients. Therefore, younger patients may have better prognoses despite the overall disappointing survival rates of SCNSL patients [15]. In this study, patients with new disease possessed higher CR rates than relapse and refractory patients (70% vs 50% and 40%). We postulated that this is because patients with relapse and refractory disease were more likely to be both drug resistant and develop treatment toxicity. This would indicate that SCNSL patients require more individualized treatment regarding their disease condition and regimen prior to the onset of CNS disease.

Benefits from the use of rituximab for PCNSL are controversial [20]. A meta-analysis published in 2019 that included two randomized control studies concluded that rituximab could improve PFS but did not significantly improve OS (hazard ratio 0.76; 95% CI 0.52–1.12, low certainty). Nevertheless, the majority of research on SCNSL treatments included rituximab (some as part of the peripheral lymphoma treatment) [10–12, 15, 17]. El-Galaly et al. concluded that rituximab may reduce the risk of death in patients with isolated SCNSL; they also emphasized that in this subgroup, rituximab can even play a role comparable to ASCT [4]. Thus, we also included rituximab as part of our treatment for SCNSL.

The present study had a few limitations. First, it is a single neurosurgery center retrospective study, selection bias may exist. Second, owing to the rarity of this disease, the sample size was small, and there is inconsistency in lymphoma type. Third, the study did not reach median OS. Prospective studies with larger sample size and longer follow-up were needed in the future.

## Conclusions

Treatment of SCNSL has always been challenging. This single-center study indicate that the R-MIADD regimen is effective in treating SCNSL and the outcome of patients receiving R-MIADD were comparable to previous reports of ASCT-containing regimens. Larger cohort prospective studies are needed to define the most effective treatment strategies for SCNSL.

## Abbreviations

ASCT: Autologous stem cell transplantation
CNS: Central nervous system
CR: Complete remission
CT: Computed tomography
DLBCL: Diffuse large B cell lymphoma
DP: Disease progression
MTX: Methotrexate
MRI: Magnetic resonance imaging
ORR: Overall response rate
OS: Overall survival
PCNSL: Primary central nervous system lymphoma
PFS: Progression-free survival
PR: Partial remission
SCNSL: Secondary central nervous system lymphoma
WBRT: Whole brain radiotherapy
